# A multicenter experience using adipose-derived mesenchymal stem cell therapy for cats with chronic, non-responsive gingivostomatitis

**DOI:** 10.1186/s13287-020-01623-9

**Published:** 2020-03-13

**Authors:** Boaz Arzi, Santiago Peralta, Nadine Fiani, Natalia Vapniarsky, Nopmanee Taechangam, Ubaldo Delatorre, Kaitlin C. Clark, Naomi J. Walker, Megan R. Loscar, Milinda J. Lommer, Amy Fulton, Jean Battig, Dori L. Borjesson

**Affiliations:** 1 Department of Surgical and Radiological Sciences, University of California, One Shields Avenue, Davis, CA 95616 USA; 2 Veterinary Institute for Regenerative Cures, School of Veterinary Medicine, University of California, Davis, CA 95616 USA; 3 Department of Clinical Sciences, College of Veterinary Medicine, Cornell University, Ithaca, NY 14853 USA; 4 Department of Pathology, Microbiology and Immunology, School of Veterinary Medicine, University of California, Davis, CA 95616 USA; 5 William R. Pritchard Veterinary Medical Teaching Hospital, University of California, Davis, CA 9516 USA; 6 Aggie Animal Dental Center, Mill Valley, California USA; 7 Animal Dental Clinic, Lake Oswego, OR 97035 USA

**Keywords:** Multicenter, Shipped adipose-derived stem cells, Fresh, Allogeneic, Autologous, Cats, Gingivostomatitis, Oral mucosa, Immunomodulation

## Abstract

**Background:**

The ability of mesenchymal stem cells (MSCs) to modulate immune responses inspired a series of clinical trials addressing oral mucosal inflammation. We previously reported on the safety and efficacy of fresh, allogeneic and autologous, adipose-derived mesenchymal stem cells (ASCs) to treat feline gingivostomatitis (FCGS), an oral mucosal inflammatory disease that shares similarities with human oral lichen planus.

**Methods:**

To meet clinical demand and goals for future commercialization, we determined the feasibility of shipping fresh ASCs to distant clinics and extended our pilot studies to expand safety and efficacy data for shipped and non-shipped ASCs in a cohort of 18 FCGS cats enrolled locally and at a few different locations within the USA.

**Results:**

We found that ASCs retained their viability, phenotype, and function after shipment. ASCs administered systemically resulted in a 72% positive response rate, identical to that noted in our previous studies. Cats that responded to ASC therapy had a significant decrease in circulating globulin concentration and histological evidence of decreased CD3+ T cells and CD20+ B cells in the oral mucosa. Responder cats also had significantly decreased percentages of CD8^lo^ cells in blood prior to and at 3 months post-ASC therapy. CD8^lo^ cells may serve as a potential “predictor” for response to systemic ASC therapy.

**Conclusion:**

Fresh feline ASCs can be successfully shipped and administered to cats with FCGS. ASCs modulate the immune response and demonstrate efficacy for chronic oral mucosal inflammatory lesions that are characterized by CD8+ T cell inflammation and T cell activation. FCGS is a potentially useful naturally occurring large animal model of human oral inflammatory diseases.

## Background

Stem cell therapy is emerging as an approach to treat patients with chronic inflammatory disorders, and adipose-derived mesenchymal stem cells (ASCs) are currently a favored cell type used in clinical trials [1]. ASCs have a profound regenerative capacity and the ability to modulate both innate and adaptive immunity resulting in potent anti-inflammatory effects [1–8]. Although the mechanism(s) of immunomodulation remain incompletely understood, ASCs can decrease cytokine release from and suppress proliferation of activated T cells. In addition, the administration of mesenchymal stem cells (MSCs) can increase regulatory T cell numbers and dampen cytotoxic T cell attack on foreign cells or tissues [9]. MSCs can also alter B cell functions, downregulate MHC II expression, and inhibit dendritic cell maturation and differentiation [3, 4, 6, 7, 10]. MSCs inhibit the mitogen-induced response to naïve T lymphocytes, CD4+ and CD8+, and natural killer cells [11, 12]. In addition, MSCs require licensing with interferon gamma (IFN-γ) to exert their immunosuppressive effects [13]. As such, MSC therapy is being explored for the treatment of immune-mediated diseases.

In cats, in vitro data support the use of ASCs for diseases associated with T cell activation. ASCs inhibit T cell responses via the secretion of soluble factors and/or direct cell-to-cell interactions. Feline ASCs decrease activated T cell proliferation and decrease tumor necrosis factor alpha (TNF-α) secretion [14]. As with other species, IFN-γ is produced by both CD4+ and CD8+ T cells upon mitogen activation; however, specifically for feline ASCs, IFN-γ production is enhanced by T cell-ASC contact [14]. This contact is mediated by ASC ICAM-1 (inducible cell adhesion glycoprotein) interacting with the T cell ligand CD11a/CD18 (β2 integrin) with resultant inhibition of T cell proliferation via cell cycle arrest in G0–G1 [14]. These findings deepen our understanding of how feline ASCs modulate T lymphocyte activity.

Client-owned pets often have naturally occurring diseases that reflect the complexity of human diseases. Data from these naturally occurring large animal models can be used to inform best practices for human clinical trials and can serve as relevant large animal data for investigational new drug applications [15–17]. Discovery of therapeutics that works for naturally occurring diseases in companion animals can drive human clinical trials [18].

Feline chronic gingivostomatitis (FCGS) is a naturally occurring immune-mediated oral mucosal disease potentially triggered by a viral etiology such as feline calicivirus [19–22]. Due to marked similarity in clinical presentation, histological manifestation, and local and systemic immune response, FCGS is a candidate for a large animal model for human conditions such as oral lichen planus, stomatitis, pemphigus, and pemphigoid [22–24]. In both humans and cats, these diseases result in painful inflammatory mucosal lesions that markedly affect quality of life and often require long-term immunosuppressive therapy. To that end, our group has reported on the efficacy of autologous and allogeneic ASCs in the treatment of refractory FCGS in non-controlled single-center clinical trials [22, 23]. Approximately two-thirds of FCGS cats treated with two doses of 20 × 10^6^ intravenously (IV) administered ASCs responded well to treatment and demonstrated cure or substantial clinical improvement with no relapse.

Successful single-center clinical trials are insufficient to demonstrate widespread therapeutic efficacy. They provide a limited ability to predict the therapeutic utility of investigational new drugs [25–27]. Successful therapies, identified in single-center clinical trials, can fail to meet efficacy standards in multicenter clinical trials [25, 26, 28, 29]. For this reason, the Federal Food and Drug Administration mandates multicenter clinical trials before drug approval. In this work, we transitioned from a single- to a multicenter non-randomized clinical trial to determine the feasibility of ASC therapy for the treatment of refractory FCGS in a larger and more heterogeneous patient population.

The first objective of this study was to determine if feline ASCs could be shipped via a commercial courier and retain their phenotype and potency after 24 or 48 h of shipment. Our second objective was to increase our safety and efficacy data in cats treated both locally and in a small cohort of cats treated at distant locations. These data serve only as a proof of concept for a larger, planned multicenter clinical trial. Finally, we enrolled a small cohort of FCGS cats for up to 6 months prior to initiating ASC therapy to serve as their own controls. These cats served to document that the hematologic, immunomodulatory, and clinical improvements we observed after ASC therapy do not occur in the absence of ASC administration. We hypothesized that (1) feline ASCs would maintain their viability, phenotype, and potency following shipment; (2) ASC therapy in cats at distant locations would be safe; and (3) in the absence of ASC therapy, FCGS would not resolve and associated hematologic and biochemical abnormalities would not change. These data will serve as a proof of concept for a larger, multicenter clinical trial. We enrolled cats at 2 academic and 2 private institutions and found that ASCs administered systemically within 24 h of shipment resulted in a 72% positive response rate, comparable to our previous results.

## Materials and methods

### In vitro shipping study

#### ASC isolation, expansion, and storage

Feline ASCs from 5 individual donors were thawed and expanded exactly as previously described [22]. Our shipping study included ASCs derived from cats with FCGS (*n* = 3) and ASCs derived from specific pathogen-free (SPF) cats (*n* = 2) to determine shipment parameters for both autologous and allogeneic ASCs. Cats enrolled in the clinical trial were later administered these same SPF-derived ASCs. After washing, 20 million ASCs were re-suspended into 2 mL of lactated Ringer’s solution and aliquoted into Class B Clear Glass Threaded Vials (Fisherbrand™, Thermo Scientific, Ottawa, ON). Vials were packaged upright and placed into Thermosafe® Insulated Shippers and a shipment simulation was conducted. Temperature was monitored using a LogTag® TRIX-8 Recorder packaged within the shipper. Temperature data was analyzed with LogTag® Analyzer software. Cells were shipped at 4 °C via FedEx commercial courier.

#### ASC characterization of phenotype and potency following shipment

ASC number (cell recovery), viability, sterility, endotoxin, cell identity (CD105), purity (CD18), MHC II expression, and potency (i.e., ability to inhibit activated lymphocyte proliferation) were determined at time 0 (baseline) and at 24 and 48 h post-shipment. Shipped ASCs were replated at 5000 cells/cm^2^ to confirm cell re-adherence and viability (24 and 48 h). Viable cell number was determined using the trypan blue exclusion test and a hematocytometer. Cell viability was confirmed through 7-AAD uptake and analyzed on a flow cytometer (Cytomics FC500, Beckman Coulter, Miami, FL, USA). Sterility was determined via aerobic and anaerobic bacterial culture (University of California, Davis, Veterinary Medical Teaching Hospital, microbiology labs), and Mycoplasma PCR (MycoScope™ PCR Mycoplasma Detection Kit, Genlantis, San Diego, CA, USA). Endotoxin levels were determined (Endosafe® nexgen-PTS™ reading device, Charles River, Charleston, SC, USA). Surface protein expression for MHC II (activation), CD105 (identity), and CD18 (purity) were determined using flow cytometry as described previously [30]. All antibodies were purchased from the Leukocyte Antigen Biology Laboratory, University of California, Davis, unless otherwise indicated. Antibody clones included MHC II (42.3), CD18 (FE3.9F2), CD105 (SN6; eBioscience, San Diego, CA, USA), and a mouse IgG-phycoerythrin (PE) antibody (Jackson ImmunoResearch Labs, West Grove, PA, USA) for secondary labeling for unconjugated antibodies. The ability of shipped ASCs to inhibit activated lymphocyte proliferation was determined using a leukocyte proliferation assay according to a previously reported method [22, 23]. ASCs at time 0, 24, and 48 h post-shipment were co-cultured with allogeneic peripheral blood mononuclear cells (PBMCs) stimulated with ConcanavalinA (ConA, Sigma-Aldrich, St. Louis, MO, USA), as previously described [22, 31].

### In vivo clinical study

#### Study population

The Institutional Animal Care and Use Committees at the University of California, Davis, and at Cornell University, and the Clinical Trials Review Board of the University of California, Davis, approved this study. All cat owners signed an informed consent prior to enrollment. A total of 18 client-owned cats with refractory FCGS were recruited to the study between the years 2015 and 2018. Inclusion criteria included cats affected by FCGS with no other primary co-morbidities that had failed to respond to full-mouth extractions performed at least 6 months prior to enrollment. All systemic or topical corticosteroid or other immunosuppressive therapies were discontinued 2 weeks prior to and for the entire duration of the clinical trial. The absence of retained tooth root tips and other underlying lesions were ruled out by full-mouth radiographs. All cats were confirmed negative for feline immunodeficiency virus and feline leukemia virus infection.

#### Clinical trial design

Cats with FCGS were recruited from four institutions (University of California Davis, Cornell University, and two private dental specialty practices) that met the inclusion criteria. Blood was collected for a complete blood count, serum biochemistry profile, and blood lymphocyte phenotyping. The study was non-randomized and not blinded; however, a cohort of 6 cats was enrolled in a crossover design where they served as their own controls for 6 months prior to being enrolled in the study. Thirteen cats were treated at UC Davis, three cats at Cornell, and one cat in each of the private specialty practice (Table 1). Oral mucosal biopsies were collected prior to ASC administration and at 6 months post administration (end of study). Clinical disease severity was evaluated using a stomatitis disease activity index (SDAI) scoring system as previously described [22, 23, 32]. The SDAI scoring was performed at the time of study enrollment and at 6 months after the first ASC administration (supplemental figure). Briefly, the cats’ owners completed a questionnaire and scored appetite, activity level, grooming behavior, and perceived oral comfort on a scale of 0 to 3. Lesion severity was also scored by a veterinary dental specialist in each participating center and experienced in FCGS evaluation, as 0 (no lesion), 1 (mild), 2 (moderate), or 3 (severe). The SDAI score for each cat was calculated at each time point (range = 0, no disease, to 30, severe disease). Cats were evaluated at day 0 and at 1 month (second injection), 3 months and 6 months after the first ASC treatment (study exit). However, cats were followed up continually for up to 24 months and data presented include the final recheck as well. During the study period, the cats received only opioid analgesic management (i.e., buprenorphine or oxymorphone) without any immunosuppressive, antibiotic, or non-steroidal anti-inflammatory medication.

**Table 1:** Demographics of the cats enrolled in the ASC clinical trial including cell source and outcome

Cat #	Age (years)	Sex	Weight (kg)	Cell source	Treatment site	Response	SI entry	SI exit	Last SI	Follow-up (months)
1	6	MC	4.3	Autologous	UC Davis	Cure	19.8	–	0	18
2	5	FS	4.7	Autologous	UC Davis	No/minimal	19	19		6
3	5	MC	4.5	Autologous	UC Davis	Substantial	14.75	4		6
4	9	FS	3.3	Autologous	UC Davis	Substantial	19	12		6
5	7	FS	7	Autologous	UC Davis	Substantial	12.75	2		12
6	11	MC	5.7	Autologous	UC Davis	Substantial	23.25	7.5		6
7	3	MC	5	Autologous	UC Davis	No/minimal	24.75	15.75		6
8	9	MC	4.6	Autologous	UC Davis	Cure	23.5	0.5		6
9	4.5	FS	3.6	Allogeneic	UC Davis	Cure	24.5	–	1	12
10	6	MC	11	Allogeneic	UC Davis	No/minimal	17.12	10		6
11	10	FS	5.6	Autologous	UC Davis	Substantial	6.25	3.25		18
12	3.5	MC	3.9	Autologous	Cornell	Cure	12.75	2		24
13	4	MC	4.4	Autologous	Cornell	Substantial	11.25	11	12.7	13
14	4	FS	6.2	Autologous	Cornell	Substantial	13.25	11.25		6
15	10	MC	7.3	Allogeneic	Oregon	Substantial	19	11.75		6
16	11	FS	3.2	Allogeneic	UC Davis	Cure	24.5	0.5		6
17	4	MC	4.1	Allogeneic	UC Davis	No/minimal	8.37	6.25		6
18	4	FS	2.9	Autologous	San Francisco	No/minimal	–	–		6

#### ASC treatment

All cats received two IV transfusions of 20 × 10^6^ (~ 5 million ASCs/kg) fresh (i.e., cryopreserved ASCs that were revived in culture for ~ 72 h prior to administration) allogeneic (*n* = 5) or autologous (*n* = 13) ASCs, 1 month apart, as previously described [22, 23]. ASCs are used at passage 2 or 3. Initially, we utilized autologous ASCs. However, due to the deleterious affect of feline foamy virus on feline ASCs, our work has exclusively switched to treating FCGS cats with allogeneic ASCs [30]. Allogeneic ASCs were derived from 2 separate SPF cats. Cells for treatment were from the same master cell bank used for the in vitro shipping study. Cryopreserved ASCs were revived in culture for ~ 72 h prior to administration. Fresh ASCs were prepared and transferred to glass vials. At the University of California, Davis, the ASCs were used within 2–3 h of placing the cells in a glass vial. In the other 3 participating centers (Cornell University and two private specialty practices), the ASCs were shipped overnight using FedEx commercial delivery shipment (exactly as described for the in vitro study) and the cells were administered within 24 h of placement within the vial. All ASCs were administered by direct injection over a period of 20–60 min by dividing the total dose into 4 separate aliquots (~ 5 million cells at a time) to prevent ASC adherence to syringe plastic and to prevent reactions associated with rapid cell infusion. All cats were hospitalized for 24 h post-treatment to monitor for adverse reactions.

#### Histology and immunohistochemistry

Oral biopsies were fixed en bloc in 10% neutral buffered formalin, embedded in paraffin, sectioned, mounted, and stained with hematoxylin and eosin according to standard laboratory protocol, as previously described [33, 34]. Immunohistochemistry was performed on 4-mm-thick, formalin-fixed, paraffin-embedded tissue sections, mounted on charged slides and air-dried overnight exactly as previously described [22]. Primary antibodies and dilutions were rat anti-CD3 (clone 3–12, diluted 1:10, Leukocyte Antigen Biology Lab, UCD) and rabbit anti-CD20 (NeoMarker RB-9013-P1; 1:300; Thermo Fisher Scientific, Pittsburgh, PA, USA). A board-certified veterinary pathologist experienced in oral pathology and regenerative medicine (NV) interpreted all biopsies.

#### Bloodwork and lymphocyte phenotyping

Blood samples were collected into EDTA Vacutainers (BD Biosciences, San Jose, CA, USA) for a complete blood cell count (CBC) using an automated analyzer (Bayer ADVIA 120; Bayer Diagnostics, Tarrytown, NY, USA). Serum was isolated from whole blood collected without an anticoagulant, centrifuged (1000*g* for 10 min) and submitted for a full biochemical profile (Cobas c501; Roche Diagnostics International, Risch, Switzerland, http://www.roche-diagnostics.ch). Lymphocytes were phenotyped (CD4, CD8, CD21) exactly as previously described [22, 23].

#### Ex vivo mixed leukocyte reaction

Lymphocyte proliferative ability, in response to mitogen activation ex vivo, was determined in FCGS cats prior to and at 6 months post-ASC administration. In brief, feline PBMCs were isolated from whole blood using gradient centrifugation and activated with 5 mg/mL ConA. Cells were incubated for 4 days. The degree of lymphocyte proliferation was determined through 5-bromo-2′-deoxyuridine (BrdU) incorporation. Wells were spiked with BrdU at day 3 and then cells were collected and processed per the manufacturer’s instructions (BrdU Flow Kit; BD Biosciences) at day 4 [22, 23].

#### Statistical analyses

All data were assessed for their distribution by the D’Agostino-Pearson normality test. A Grubb’s test was used to determine if there were outliers (no outliers were detected or excluded). Parametric tests were performed only on data with Gaussian distribution. The difference within groups at different time points was determined either with repeated-measure or one-way ANOVA or Kruskal-Wallis test. The difference between 2 groups at specific time points was determined using Student’s *t* test or Mann-Whitney test. A two-tailed nonparametric Wilcoxon matched-pairs test was used for MSC potency. In all cases, *p* values < 0.05 were considered statistically significant. Statistical analysis was performed using GraphPad Prism Version 5.02 software (Graphpad Software, San Diego, CA, USA).

## Results

### ASCs successfully maintained viability, function, and phenotype after shipment

**Fig. 1:**
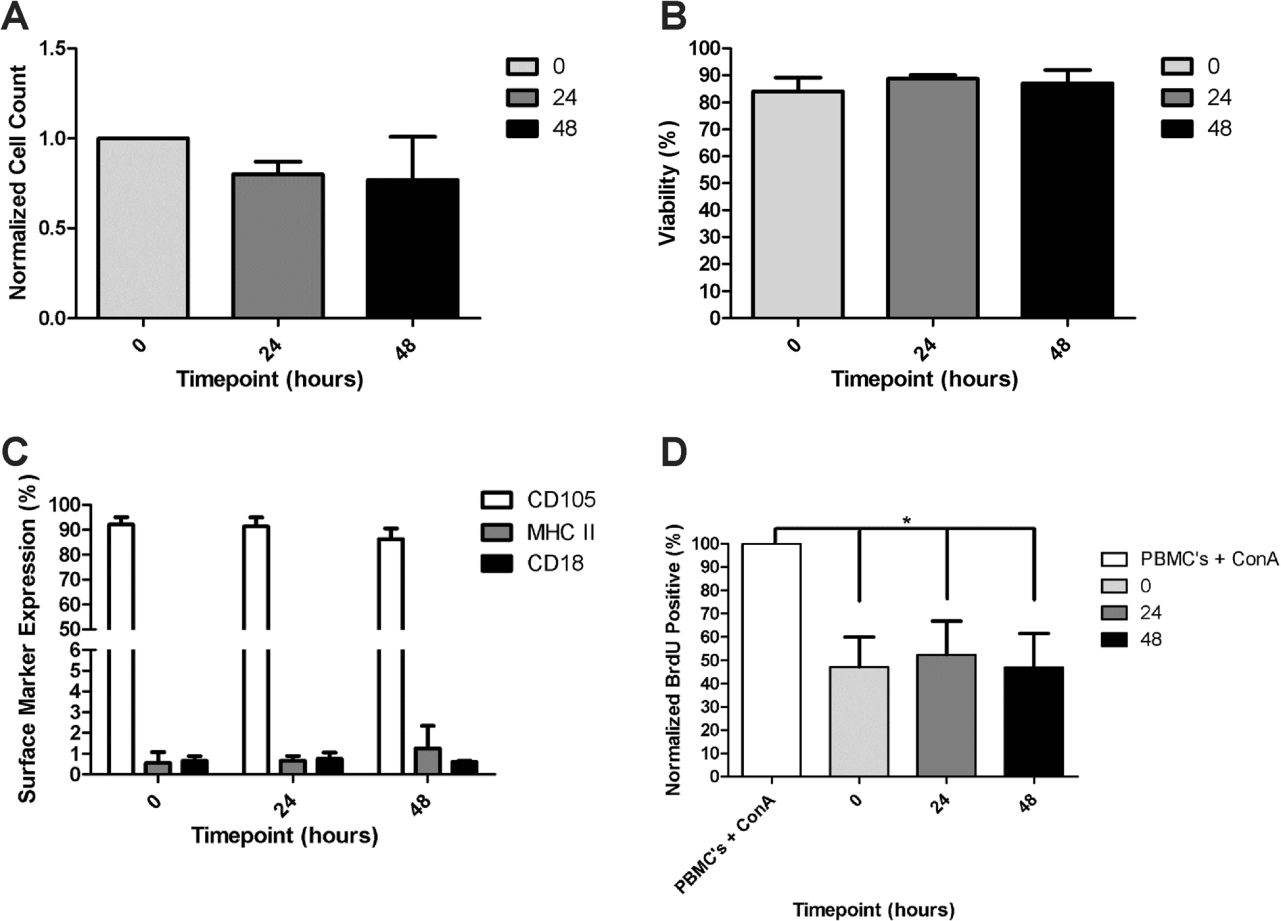
ASC characterization prior to and after shipping. **a** Normalized ASC cell counts for all three time points—0 h, 24 h, and 48 h. **b** Percent viability of ASCs at 4 °C for all 3 time points. **c** ASC surface phenotype. **d** Normalized suppression of lymphocyte proliferation for all three time points. *N* = 5 for all experiments. The % of proliferating lymphocytes was normalized to PBMC stimulated with ConA. Abbreviations: BrdU, 5-bromo-29-deoxyuridine; ConA, concanavalin A; PBMC, peripheral blood mononuclear cell

All 5 ASC cultures were successfully maintained at 4 °C throughout the shipping period (24 and 48 h). The total cell number decreased after 24 h (average 20% decrease) and after 48 h of shipment (average 24% decrease compared to baseline) although the changes were not significant (Fig. 1a). There was no reduction in cell viability over time (Fig. 1b) and all cells readily re-adhered, proliferated, and maintained normal cell morphology after re-plating. All cells at all time points were *Mycoplasma*-negative and had endotoxin levels < 5 EU/mL (safe for clinical injection). All cells were bacterial culture-negative at time 0 and at 24 h; however, at 48 h, 2 of the 5 cells were culture positive with low-level plate contaminants with non-enteric species. MHCII (MSC activation) and CD18 (MSC purity) expression was < 1% on all cells at all time points (Fig. 1c). The expression of CD105 (MSC identity) did not significantly change over time (CD105 decreased an average of 12%), remaining > 85% positive over time (Fig. 1c). All 5 ASC cultures significantly inhibited activated lymphocyte proliferation at time 0 (*p* = .03), 24 (*p* = .03) and 48 h (*p* = .04) after shipment (Fig. 1d). Given the occasional positive bacterial culture at 48 h, ASCs were administered within 24 h after placement in the vial for the multicenter clinical trial.

### Autologous and allogeneic ASCs therapy induced marked clinical improvement in cats with FCGS in shipped and non-shipped cells

**Fig. 2:**
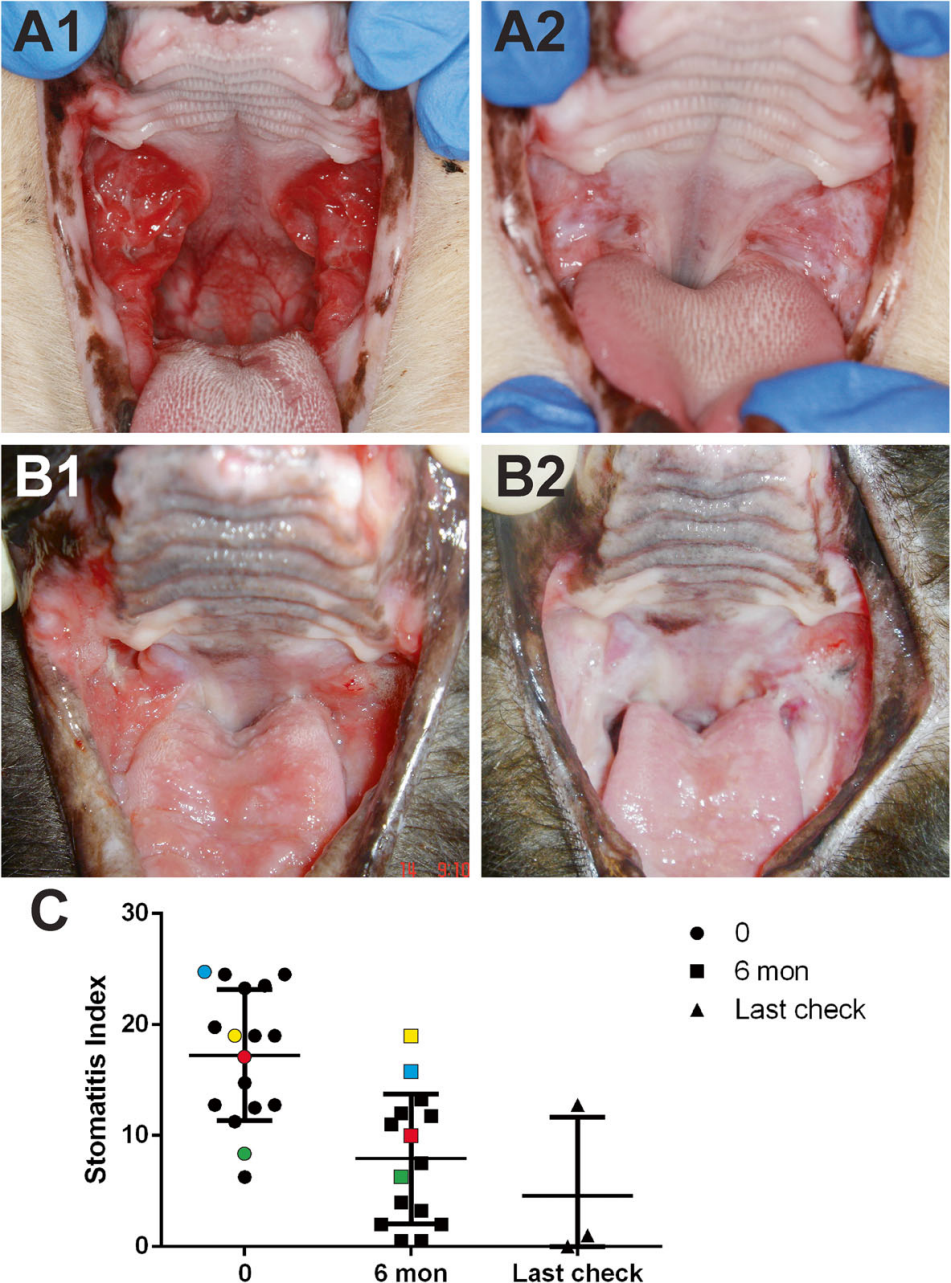
Clinical assessments of disease severity by means of clinical images and stomatitis disease activity index (SDAI) over time. Representative pre-treatment images for 2 different cats (A1, B1) are characterized by severe proliferative and ulcerative inflammation of the caudal oral cavity, the area lateral to the palatoglossal folds. Note the profound improvement with complete cure in one cat (A2) and a substantial improvement in the other (B2). In the cat depicted in B1 and B2, there was also glossitis that improved substantially. SDAI graph demonstrating the score at entry and exit examination (see Table 1) as well as the last recheck available. Non-responder cats are color-coded (blue = cat#7, yellow = cat#3, red = cat#10, green = cat#17). Abbreviation: SI, stomatitis index

Of the 18 cats that were enrolled (10 males, 8 females), 17 completed the study to 6 months (Table 1). One cat was lost to follow up after the 3 months recheck, and that cat had substantial improvement at 3 months. Of the 13 cats that received autologous cells, 77% (10/13) were categorized as having substantial improvement or cure determined by oral examination, photographs, histology, and stomatitis index. Of the 5 cats that received allogeneic cells, 60% (3/5) were categorized as having substantial improvement or cure. Breaking this down further, 5/18 cats (27.8%) responded to treatment with complete cure (Fig. 2a) and 8/18 cats (44.4%) exhibited substantial clinical improvement. Taken together, 72.2% of the cats in this study exhibited a positive clinical response to ASC treatment. The cats with substantial improvement/cure were enrolled at the University of California, Davis; Cornell University; and a private practice in Oregon. It is important to note that, as reported previously [22, 23], clinical response was observed between 3 and 6 months after ASC administration. Finally, 5/18 (27.8%) cats did not respond to treatment or had minimal improvement.

The SDAI was completed on all but one patient. Clinical assessment of disease severity, by means of the SDAI, was generally in agreement with our clinical observations (Fig. 2b, c). In general, the improvement of clinical signs corresponded with the improvement of the oral mucosal lesions. Responder cats gained weight and returned to normal eating behavior, grooming, and sociability. Cat owners reported a return to pre-FCGS activity levels in the responder cats. The 5 cats that did not respond to treatment had static or worse SDAI.

### Oral inflammation, hematologic values, and biochemical parameters did not improve over 6 months in the absence of ASC therapy

**Fig. 3:**
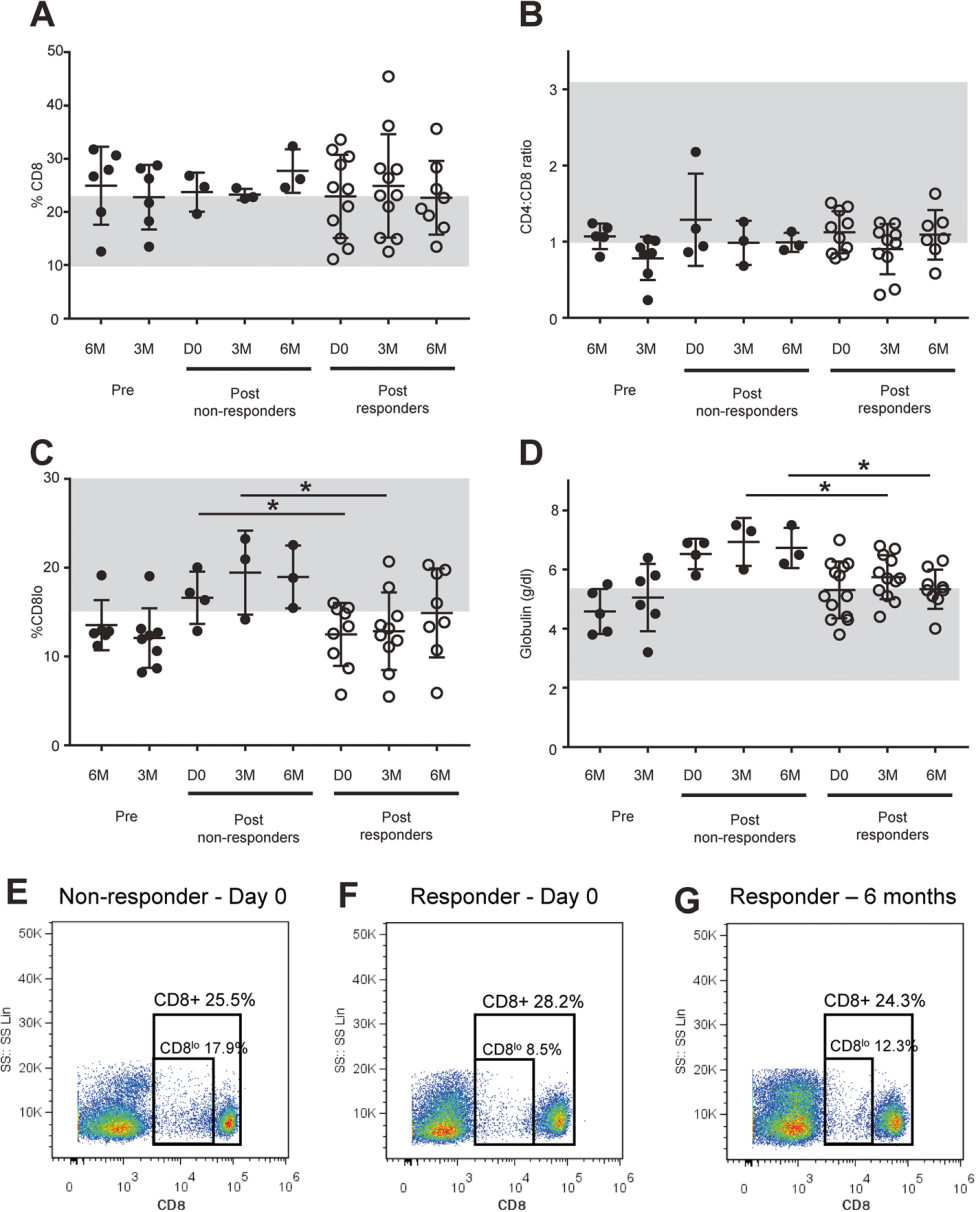
Bloodwork and PBMC proliferative response in FCGS patients receiving ASC treatment. Changes in **a** percentage of CD8+ T cells and **b** CD4+/CD8+ T cell ratio; **c** percentage of CD8+ T cell with CD8 receptor downregulation (CD8^lo^ cells) out of total CD8+ T cells; and **d** serum globulin level, measured prior to and after ASC administration. Note the significant reduction in serum globulins in responder cats corresponded to clinical improvement. Reduction in CD8^lo^ cells was also significant between pre- and post-ASC therapy. Representative flow cytometry plots for CD8+ and CD8^lo^ populations for **e** non-responder FCGS-affected cat at day 0; **f** responder FCGS-affected cat at day 0; and **g** responder FCGS-affected cat at 6-month follow-up after ASC treatment. CD8^lo^ percentages of CD8+ cells based on flow cytometry gates shown in **e**–**f** for all cats in the study. Non-responder cats had a significantly higher percentage of CD8^lo^ cells before ASC treatment (Mann-Whitney test; *p* = 0.02)

One of the goals of this study was to enroll a small cohort of FCGS cats as controls to determine if (1) cats would cure in the absence of ASC therapy and (2) the hematologic alterations that we had previously noted (i.e., CD8^lo^ cells, globulin concentration, and neutrophil number) would change in the absence of ASC therapy. We enrolled 8 FCGS cats as their own controls prior to crossing them over into the treatment group. Six of these cats (75%) were maintained for the full 6 months prior to ASC therapy; 2 of these cats were maintained for only 3 months prior to crossing them over into the treatment group due to severe progressive disease. None of the 8 cats experienced substantial disease improvement or cure in the absence of ASC therapy. There were no significant differences in percent CD8+ T cells, CD4/CD8 ratio, percent CD8^lo^ cells, or globulin concentration in the 6 months prior to ASC administration in these control FCGS cats (Fig. 3a-d).

### Cats that respond to ASC therapy have decreased globulin concentration over time and a lower percentage of circulating CD8^lo^ T cells prior to therapy

Thirteen cats received autologous ASCs and 5 cats received allogeneic ASCs. No significant differences were detected in hematologic or biochemical data between FCGS cats that received allogeneic ASCs and FCGS cats that received autologous ASCs. As such, their data were combined for all bloodwork analyses (*p* > 0.05). Cats with FCGS had a variable leukocytosis due to a neutrophilia, as well as hyperglobulinemia. Responder and non-responder cats mostly did not differ in basic white blood cell parameters, total protein concentration, or albumin concentration over time (data summarized in Table 2). Responder cats had a significantly higher neutrophil count at 3 months (*p* = 0.05, Table 2) than non-responders. However, this difference was not noted by the end of the study. Cats that responded to ASC therapy had a significantly decreased total protein concentration at 3 months due to decreased globulin concentration (*p* = 0.05, Table 2, Fig. 3d), and the significant decrease in globulin concentration was sustained at 6 months (*p* = 0.01). The majority of cats (8/14, 57%) had increased percentages of CD8+ T cells in blood prior to treatment (day 0, Fig. 3a). The percentage of circulating CD8+ T cells tended to increase over time in the non-responders, suggesting sustained cytotoxic immune activation. However, cats that responded to ASC therapy demonstrated decreasing percentages of CD8+ T cells, but the difference was not statistically significant (Fig. 3a). The CD4/CD8 ratio did not significantly change prior to or after ASC therapy regardless of response to ASC therapy (Fig. 3b). Previously, we reported on decreased percentages of CD8^lo^ T cells (at time 0) as being one potential biomarker that predicted response to ASC therapy in cats with FCGS. This finding was recapitulated for cats in this study. Compared with cats that did not respond, cats that responded to ASC therapy had significantly lower percentages of CD8^lo^ T cells at day 0 (*p* = 0.02, Fig. 3c) and at 3 months post-treatment (*p* = 0.05, Fig. 3c). Like all other parameters, the percentage of CD8^lo^ cells did not change in the 3–6 months prior to ASC therapy (Fig. 3d).

**Table 2:** Selected hematologic and biochemical parameters of FCGS patients prior to and after ASC therapy

Parameters		Non-responders (***n*** = 5)			Responders (***n*** = 13)			***p*** value
	Time point	Mean	SD	Range	Mean	SD	Range	
**White blood cell count (/**μl**)**	**Pre-treatment**	10,948	1659	8740–12,340	13,807	7410	3900–28,080	0.53
	**3 months**	8980	4158	5530–13,597	12,284	5966	6200–28,300	0.05*
	**6 months**	13,667	1527	11,230–15,270	13,652	6713	6700–29,400	0.90
**Lymphocyte count (/μl)**	**Pre-treatment**	2597	643.9	1713–3122	1969	970.5	741–3973	0.23
	**3 months**	2082	634.8	1466–2734	1986	1161	926–5452	0.19
	**6 months**	2870	1018	1771–3780	2308	1676	986–6582	0.18
**Neutrophil count (/μl)**	**Pre-treatment**	7254	1223	6092–8724	10,422	6878	2145–22,717	0.61
	**3 months**	5326	4070	2544–9998	8831	5923	2820–24,338	0.08
	**6 months**	9598	2724	7705–12,720	10,398	6859	3021–26,372	0.75
**Total protein (g/dl)**	**Pre-treatment**	9.45	0.6856	8.5–10	8.462	0.76	7.3–9.5	0.10
	**3 months**	9.6	1.127	8.3–10.3	8.6	0.6015	7.6–9.4	0.05*
	**6 months**	9.233	0.7572	8.7–10.1	8.36	0.6931	7.5–9.4	0.16
**Albumin (g/dl)**	**Pre-treatment**	2.925	0.1708	2.7–3.1	3.108	0.5664	2.3–4.1	0.71
	**3 months**	2.667	0.3215	2.3–2.9	2.867	0.3339	2.4–3.6	0.99
	**6 months**	2.5	0.2646	2.2–2.7	3	0.3742	2.4–3.7	0.27

### ASC therapy results in decreased lymphocyte proliferative ability in the majority of responder cats

**Fig. 4:**
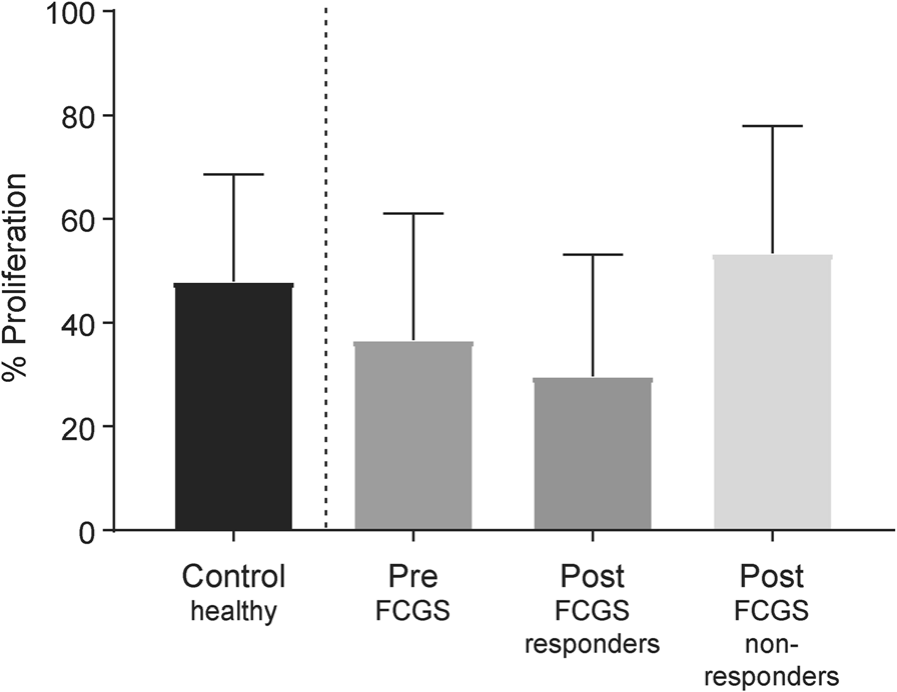
The proliferative response of PBMCs from FCGS patients prior to and after treatment with ASCs as determined through BrdU incorporation. Sample sizes (*n*), 3–6 cats for non-responder group and 11–13 cats for responder group; pre, prior to ASC treatment, post, after ASC treatment, D0, day 0; 3M, 3-month follow-up; 6M, 6-month follow-up

The ability of lymphocytes from FCGS patients to proliferate in response to mitogen activation was tested prior to and at 6 months post-ASC administration. At baseline, lymphocyte proliferation in FCGS cats was comparable to healthy control cats (Fig. 4). At 6 months post-ASC therapy, 75% of the cats that responded to therapy had decreased lymphocyte proliferative ability compared to their baseline. On the contrary, 80% of the cats that did not respond to therapy at 6 months demonstrated comparable or greater proliferative capability as compared to their baseline. Due to marked individual variation in absolute lymphocyte proliferative response, the data between responders and non-responders were not statistically different (Fig. 4).

### Histopathologic features correlated with clinical findings in cats receiving shipped and non-shipped ASCs

Oral mucosal biopsies were obtained from all cats prior to study enrollment and at 6 months post-ASC treatment. The histopathological features of the present study were identical to our previous reports [22, 23, 33]. In all pretreatment biopsies, the epithelium and sub-epithelial stroma were expanded by a mixed inflammatory infiltrate composed of lymphocytes, plasma cells, and neutrophils, with occasional Mott cells, mast cells, and histiocytes. Ulceration of the surface epithelium was frequently observed. Remnant surface epithelium was hyperplastic with multiple rete pegs extending deep into the subjacent stroma. Immunohistochemistry revealed that CD3+ T cells were present within the epithelium and sub-epithelial stroma, while CD20+ B cells were restricted to the sub-epithelial stroma. The biopsies obtained from the non-responder cats at 6 months post-treatment were similar to the pre-enrollment biopsies. In the cats that demonstrated substantial clinical improvement, a profound reduction of inflammation was observed histologically: occasional lymphocytes were observed within the sub-epithelial stroma, with no evidence of epithelial hyperplasia, ulceration, or inflammation. In cats that exhibited cure, a complete return to normal tissue architecture with no inflammatory cell infiltrates was observed (data not shown).

### Adverse effects

Four cats (1 at the University of California, Davis; 2 at Cornell University; and 1 at a private practice) developed edema in the forelimb that was used for IV ASC administration. For two cats, the IV catheter was removed, diphenhydramine was administered, and the edema resolved within several hours with no further consequences. One cat developed skin necrosis, experienced prolonged skin recovery, and underwent skin grafting. The cat is now doing well. For the 2 cats that developed edema of the forelimb at the first ASC administration, a central line was placed for the second dose. Two of the above cats also experienced an increase in respiratory rate during treatment that lasted several hours and also resolved spontaneously, and two additional cats experienced vomiting and diarrhea immediately after the treatment, which resolved spontaneously within a few hours. Apart from these non-life-threatening complications, no other side effects were noted.

## Discussion

In this study, we report our first multicenter experience using shipped and non-shipped ASCs for the treatment of a chronic, naturally occurring oral mucosal disease in a large animal model. First, we found that ASCs successfully maintained viability, function, and phenotype during shipment with a commercial courier at 4 °C for at least 48 h. Importantly, regardless of the cell source (allogeneic or autologous), ASC therapy induced marked clinical improvement in cats with FCGS even after shipment. Similar to our previous work, cats with a decreased percentage of CD8^lo^ T cells were more likely to respond to ASC therapy [22]. Finally, cats that responded to therapy had a substantial reduction in oral mucosal inflammation and in systemic inflammation, as evidenced by decreased globulin concentration. The majority of responder cats also had decreased lymphocyte proliferative ability ex vivo at the end of the study compared to day 0. The systemic administration of ASCs was generally safe, although some adverse events were noted in 6 cats.

MSCs are currently one of the most used cells in pre-clinical and clinical trials in humans and animals [35, 36]. However, there is substantial variability in clinical outcomes that limit MSC use and regulatory approval. Discrepancies in clinical outcomes have been attributed to cell culture, expansion and storage protocols, and cell source as well as the method of transportation [37, 38]. Specifically, MSC viability and function may be altered in the time between processing at the laboratory and administration to the patient. Hence, cell transport requires precise evaluation and control of the temperature and shipping conditions to optimize safety and efficacy [39]. In the first part of the study, we found that fresh ASCs successfully maintained viability, function, and phenotype after shipment with a commercial courier at 4 °C for up to 48 h. These data agree with previous studies of human bone marrow-derived MSC storage that demonstrated satisfactory viability for 24 h at 4 °C [40, 41]. Our data suggest that shipment in lactated Ringer’s for up to 48 h would be acceptable other than the low level of bacterial contamination noted. ASC lines cultured in clinical laboratories, even under good manufacturing practices or good laboratory practice protocols, can have low-level bacterial growth using standard microbiology techniques (usually a contaminant). This is not an issue limited to MSC culture but rather is a long-standing issue in all cell therapy fields [42]. Regardless, our current recommendation for feline ASC lines that are processed and shipped from our clinical laboratory is that the cells be administered within 24 h of processing.

Chronic inflammatory oral mucosal diseases such as FCGS and oral lichen planus and stomatitis in humans are thought to have a complex and heterogeneous pathogenesis. It is believed that FCGS is triggered by or associated with feline calicivirus, but the exact etiology remains elusive [19, 20]. Regardless, FCGS and oral lichen planus have several commonalities: both are chronic inflammatory diseases affecting the oral mucous membranes, both are T cell-mediated diseases in which CD8+ cytotoxic T cells predominate in affected tissues, and T cells (mostly CD8+ and some CD4+ cells) migrate from the systemic circulation to the mucosal epithelium. Both diseases are also associated with elevated serum levels of globulins, primarily IgGs [33, 43–45].. Our data suggest that IV administration of fresh ASCs can modulate these features of immune-mediated disease. Disease improvement or resolution of clinical signs was associated with a decreasing percentage of circulating CD8+ T cells and a significant reduction in globulin concentration. Finally, in the ex vivo mixed lymphocyte reaction, lymphocytes from the majority of cats that responded to ASC therapy were less proliferative to mitogen at 6 months (compared to their own baseline), whereas lymphocytes from the majority of cats that did not respond to ASC therapy were as or more responsive to ex vivo stimulation at 6 months. Together, these data support potent immunomodulatory effects post-MSC administration [46, 47].

We previously reported on the presence of decreased percentages of CD8^lo^ T cells as being one potential biomarker for predicting a response to ASC therapy in cats with FCGS. Similarly, cats in this study that responded to ASCs had significantly lower CD8^lo^ T cells than cats that did not respond to therapy. Although some cats increased the number of CD8^lo^ cells in association with disease improvement, there was no real trend in CD8^lo^ cells changing with therapy in responder or non-responder cats. CD8^lo^ cells are associated with viral infections in cats, humans, and mice [48–50]. CD8^lo^ cells may represent a subset of activated CD8 effector/suppressor cells capable of downregulating the activation of naïve T cells. A decreased percentage of CD8^lo^ cells may imply that responder cats have decreased suppressor function (or less tolerogenic CD8 T cell subsets).

Regardless of the cell source (autologous or allogeneic), ASCs administered systemically induced marked clinical improvement in cats with FCGS achieving complete resolution or a substantial reduction in oral mucosal inflammation in 72.2% of the cats. These findings are similar to our previous reports on smaller cohorts of cats [22, 23]. Clinical response to therapy generally took 3–6 months, as has been observed in our previous studies, and clinical response/cure has been permanent [22, 23]. While we cannot assert the reason for the delayed response, it is plausible that ASCs are acting through systemic mechanisms that induce immune cell senescence, prevent the activation of new T cells, or provide regulatory signals to downregulate cells. In these scenarios, a clinical response would only be visible after a substantial number of pathogenic cells became exhausted or underwent apoptosis [51–53]. This is clinically relevant as it can be difficult to assure protocol compliance (client and clinician) when the response to therapy is delayed.

For this study, we enrolled 8 cats to serve as their own controls prior to cell administration (crossover design) to determine if the clinical response and hematologic parameters, especially CD8 and CD8^lo^ cell counts, would change in the absence of therapy. In our control cats, we found no evidence of improvement in clinical disease or alterations in hematologic data in the absence of therapy. These data are compatible with the fact that spontaneous recovery from refractory FCGS has never been reported [54].

Adverse reactions, related to swelling and edema of the leg that was used for systemic administration of ASCs, were noted in 4 cats. IV ASC administration to cats with FCGS results in immediate adherence of some ASCs to the vein close to the site of administration, with the majority of cells retained in the lungs, and a small proportion of ASCs trafficking to the inflamed oral mucosa [23]. Swelling and edema could be related to microscopic cell clumping. ASCs are large, and cell size and diameter are major determinants of vascular obstruction and complications emerging from it, such as the limb edema noted here [55, 56]. Furthermore, IV transplantation may cause microembolism [57]. Local microembolism can cause aggravated mechanical vascular obstruction and resultant edema and swelling. In addition, the cephalic vein that was used for administering ASCs in cats is fairly small and using a larger vein such as the jugular (i.e., a central line) may be a safer option. In 3 cats, once the catheter was removed and the cat was motivated to change position, the edema resolved within a few hours. The serious complications of leg edema and resultant skin necrosis seen in one cat suggest that mild swelling and/or edema should be promptly evaluated and addressed.

## Conclusion

In summary, we show that feline ASCs successfully maintain their viability, function, and phenotype during shipping with a commercial courier. Furthermore, we demonstrate that in a larger cohort of cats and in a small multicenter setting, feline ASCs administered systemically resulted in favorable clinical, histologic, and systemic response in over 70% of cats. Substantial resolution or cure of FCGS also resulted in a significant reduction in serum globulins and, for most cats, a decreased ex vivo lymphocyte proliferative response. Decreased CD8^lo^ cells may serve as a potential “predictor-biomarker” for the likelihood of response to systemic ASC therapy. Harnessing FCGS as a potentially useful naturally occurring large animal model, we demonstrate the clinical potential of ASC’s immunomodulatory function for therapy of chronic oral mucosal inflammatory lesions that are characterized by CD8+ T cell inflammation and T cell activation. The encouraging results of this study are potentially translatable for the treatment of human oral inflammatory diseases.

## Supplementary information

**Additional file 1.** Initial evaluation form: Stomatitis Disease Activity Index.

## Data Availability

The data that support the findings of this study are available on request from the corresponding author.

## Abbreviations

ASC: Adipose-derived mesenchymal stem cell
BrdU: 5-bromo-2′-deoxyuridine
CBC: Complete blood cell count
ConA: ConcanavalinA
FCGS: Feline gingivostomatitis
IFN-γ: Interferon gamma
IV: Intravenously
MSC: Mesenchymal stem cell
PBMC: Peripheral blood monocular cell
SDAI: Stomatitis Disease Activity Index
SPF: Specific pathogen free
TNF-ɑ: Tumor necrosis factor alpha
